# Laser Powder Bed Fusion Process Parameters’ Optimization for Fabrication of Dense IN 625

**DOI:** 10.3390/ma15165777

**Published:** 2022-08-21

**Authors:** Alexandru Paraschiv, Gheorghe Matache, Mihaela Raluca Condruz, Tiberius Florian Frigioescu, Laurent Pambaguian

**Affiliations:** 1 National Research and Development Institute for Gas Turbines COMOTI, 220D Iuliu Maniu Avenue, 061126 Bucharest, Romania; 2 Section IX-Materials Science and Engineering, Technical Sciences Academy of Romania, 030167 Bucharest, Romania; 3 European Space Research and Technology Centre (ESA-ESTEC), Mechanical Department, European Space Agency, 2200 AG Noordwijk, The Netherlands

**Keywords:** IN 625, AM, PBF-LB, density, balling, process parameters

## Abstract

This paper presents an experimental study on the influence of the main Laser Powder Bed Fusion (PBF-LB) process parameters on the density and surface quality of the IN 625 superalloy manufactured using the Lasertec 30 SLM machine. Parameters’ influence was investigated within a workspace defined by the laser power (150–400 W), scanning speed (500–900 m/s), scanning strategy (90° and 67°), layer thickness (30–70 µm), and hatch distance (0.09–0.12 µm). Experimental results showed that laser power and scanning speed play a determining role in producing a relative density higher than 99.5% of the material’s theoretical density. A basic set of process parameters was selected for generating high-density material: laser power 250 W, laser speed 750 mm/s, layer thickness 40 µm, and hatch distance 0.11 mm. The 67° scanning strategy ensures higher roughness surfaces than the 90° scanning strategy, roughness that increases as the laser power increases and the laser speed decreases.

## 1. Introduction

Additive Manufacturing (AM) is currently one of the most studied technologies that can be used to produce new high-complexity parts, and its industrialization is currently under discussion, especially due to the increase in maturity.

Since the development of AM, many methods have been improved for different raw materials, and many are available to produce high-end products. The most challenges encountered in the field of AM are related to the production of metallic parts for highly demanding industries such as space and aerospace. One major challenge of all AM methods for developing metallic parts as candidates for space applications is to produce denser materials with reduced porosity and anisotropy levels than conventional manufacturing routes [1,2]. Powder bed methods are used to produce metallic parts with characteristics mainly influenced by the feedstock properties (like shape, surface morphology, size distribution that affect the flowability of powder, and porosity) and process parameter selection [1].

Two types of defects result in metallic parts produced using powder bed methods, feedstock-induced defects and process-induced defects. Powder porosity can result in a high degree of pores in AM-produced materials [3], but the manufacturing route mainly controls the metallic powder properties and particular powders were developed and are commercially available.

However, the most common defects result from laser–material interactions [4,5] and establishing a proper combination of process parameters is a more challenging activity. Typical process parameters in metallic PBF-LB processes are powder layer thickness, beam focus, laser speed, laser beam power, hatch distance, and scanning strategy [1]. Process parameter combinations can result in defects like pores, lack of fusion (LOF), surface roughness, microfractures, delaminated areas, and part dimensional inaccuracies [6,7,8]. Based on the research available regarding the production of AM parts, the need to tailor the process parameters for each metallic material as well as for each manufacturing equipment is obvious.

Many authors [9,10,11,12,13,14,15,16,17,18,19] have studied the influence of process parameters on metallic material’s properties and microstructure, and some of them are referred to as a key-parameter combination such as volumetric energy density (EV or VED) or planar energy density (EP or ED) to define which combination of parameters should be used to obtain high-density materials (low porosity degree) ensuring high manufacturing rates. These key-parameter combinations can be calculated on the bases of Equations (1) and (2) [20,21]:

VED = P/vdt [J/mm^3^] (1)

ED = P/vd [J/mm^2^] (2)

where: P—laser power; v—scanning/laser speed; d—hatch distance; t—layer thickness.

Based on Equations (1) and (2), it can be stated that the energy density can be increased by increasing the laser powder and by decreasing the scanning speed, hatch distance, or layer thickness. The planar energy density can be used when the layer thickness is kept constant [7,22]. Even if some authors use the energy density for their process parameter optimization, others maintain it can be used in a narrow band of applicability due to its inability to capture the complex physics of the melt pool [20]. Therefore, it can be used to determine how much energy is transferred to the powder bed. Another solution would be to modify the equation to include more process parameters and material characteristics [9].

Many studies have been performed regarding the optimization of process parameters and their influence on the quality of additive manufactured parts from IN 718 or IN 625, but many focused on the influence on the material’s mechanical properties [23,24,25,26,27]. Although mechanical performance is crucial, density and surface finish should be carefully inspected, especially in industrial fields such as aerospace, where the highest manufacturing standards regarding part quality are imposed. In the as-built state, additive manufactured metallic parts are characterized by a high surface roughness due to the structure’s building angle, which has a stair-stepping effect or balling effect as spherical particles are separated from the unstable melt pool [1,6,7,28,29,30]. In their extensive paper, DebRoy et al. [1] state that the material’s performance can be affected by the roughness degree. The roughness is a result of the physical processes that govern the shape and stability of the melt pool. The process parameters’ combination affect these physical processes resulting in balling formation [6] or LOF defects. Balling appearance has also been reported to be influenced by the atmosphere; it could be reduced in atmospheres with low oxygen content (<0.1% O_2_) and it could increase in high oxygen content atmospheres (>2% O_2_) [30]. In addition to the atmosphere, the shape of the balls is an important aspect, it affects the wettability of the melt pool formed. An increase in layer thickness can result in larger balls, regardless of the hatch distance, which does not impact the balling formation [30]. Process parameter combinations can affect the integrity of deposited layers due to improper penetration and distribution, causing layer delamination and LOF defects; hence, the density and surface quality of the material is affected.

Defect emergence in additive manufactured metallic parts affects the material’s densification level. Relative densities between 95.5–99.99% were reported for IN 625 manufactured using different advanced methods [21,31,32,33,34,35,36]. For example, Marchese et al. [21] studied the densification of IN 625 additive manufactured by selective laser melting (SLM) and laser metal deposition (LMD). In their study, densification was assessed on the bases of porosity measurements on SEM images, which is not the most accurate method that can be applied, but they reported densification degrees up to 99.96% by SLM, and 99.89% by LMD were reported. Gao et al. [31] reported a relative density of 99.7% for electron beam melting (EBM) manufactured IN 625, and Terris et al. [32] obtained relative densities ranging between 95.5–99.8% for SLM manufactured IN 625 using different VED values. High relative densities of laser powder bed fusion (LPBF) manufactured IN 625 were also obtained by Wong et al. [33] and by Poulin et al. [34]. Wong et al. [33] reported a 99.99% relative density, while Poulin et al. [34] obtained the same relative densities for the same specimens, 99.74% relative density determined using the Archimedes method and 99.92% by CT scanning.

Additive manufacturing of metallic materials is considered a time-consuming technology as many trials are made until the part’s proper characteristics are achieved. Even if the process parameters are the same for several AM equipment, the specific combination of parameters for each machine and each material must be experimentally determined. A crucial activity before starting the manufacturing of metallic parts is the establishment of a specific set of parameters that ensure different targeted properties. Therefore, when a part with imposed properties is manufactured, the manufacturer will know which combination of parameters will apply, reducing the failure risk and cost associated with it.

The goal of this study was to optimize the PBF-LB process parameters to manufacture high relative density IN 625 superalloy using a Lasertec 30 SLM machine and to assess the influence of different process parameters on the material’s quality, focusing on density and surface roughness. The experiments were designed as a progressive analysis consisting of three iterations. First, the influence of laser power and scanning speed on the surface quality and densification were evaluated (process parameters initial optimization). Based on the results obtained during the initial parameters’ optimization, other two consecutive iterations were made as a process parameters optimization fine-tuning. For two laser powers, the influence of layer thickness and hatch distance on the densification of IN 625 was assessed. The most commonly used scanning strategies (90° and 67°) were used throughout the assessment. The bibliographic study showed that some authors used the Archimedes’ method to determine the material’s density, while others used microscopic methods or micro-CT. In this study, Archimedes and microscopic methods were used for density measurements to assess if a correlation between the two methods can be found. Moreover, the experimental results are discussed as a function of the individual parameters like laser power and laser speed, as well as a function of VED.

## 2. Materials and Methods

### 2.1. Specimen Manufacturing

**Figure 1:**
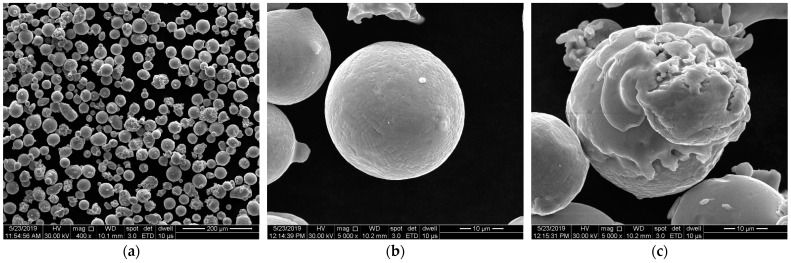
SEM images with IN 625 powder with a high degree of spheroidization (**a**), characterized by almost spherical particles (**b**) and also by satellite particles (**c**).

Vacuum gas atomized virgin IN 625 metal powder supplied by LPW Technology Ltd., a subsidiary of Carpenter Technology Corporation, was used to produce specimens for this work. The metal powder exhibits mainly spherical powder particles within the size range of 15–45 μm and particle size distribution of D_10_ = 20 ± 2 μm, D_50_ = 30 ± 5 μm, D_90_ = 45 ± 5 μm (powder size range and size distribution were provided by the manufacturer in the batch test certificate). Figure 1 shows SEM images with the powder particles. In these images, the powder morphology can be observed.

**Figure 2:**
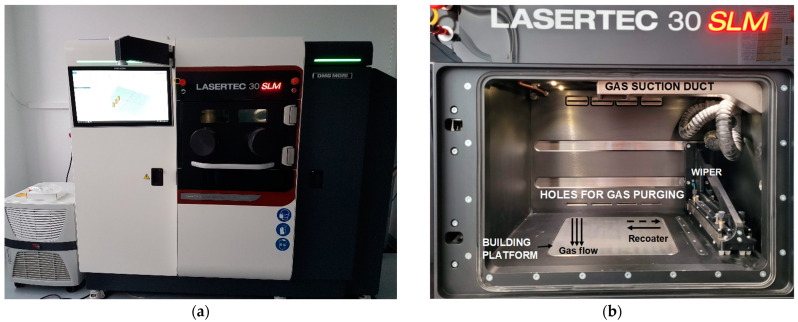
Lasertec 30 SLM machine (**a**) and the building chamber (**b**).

Prismatic specimens were manufactured using a DMG MORI Lasertec 30 SLM machine (first generation) for the experimental procedure. The Lasertec 30 SLM machine operates in a controlled environment (temperature 24 ± 2 °C and humidity < 60%). It has a ytterbium fibre laser and uses the same manufacturing principle as other powder bed machines with the feature that it has a re-plug powder module that includes a sieving unit in a closed loop for metal powder feeding and recirculation. The powder is supplied from the main tank through a sieving system and then to a secondary tank from where a lift supplies the powder into the building chamber. The metallic powder is supplied by the lift and is spread on the building plate by a rubber wiper. The machine has a building volume of 300 × 300 × 300 mm^3^ and an Ar gas flow maintains a low oxygen level within the building chamber. Before starting the manufacturing process, the building chamber is flooded with Ar to reduce the oxygen to as low as 0.2%, which is maintained all over the manufacturing process. The flow system prevents material oxidation, reduces the smoke from the building chamber and removes the condensate produced during the powder melting. The Ar is purged through four holes placed at the back of the building chamber and is suctioned through a large slot placed in the equipment’s door that is connected to the suction duct. Images of the equipment and the building chamber are presented in Figure 2. The wiper spreads the powder from right to left and afterward, it returns to the initial position. The gas flow was transverse to the direction of the powder spread (Figure 2b).

Specimens of 10 × 10 × 20 mm^3^ in size were built in the vertical position, tilted with 5° in the X-Y plane, on a stainless-steel building plate heated at 80 °C to reduce the thermal stresses in the specimens by decreasing thermal gradients during the first layer’s deposition. All specimens were built under an argon atmosphere (keeping the oxygen content at a level under 0.2%) using the same support material configuration. All specimens were manufactured without contour.

**Figure 3:**
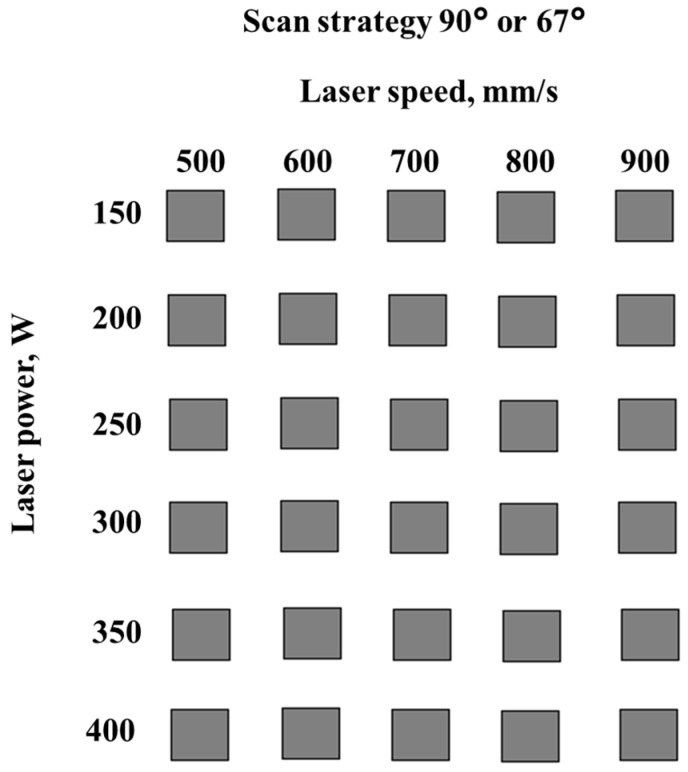
Workspace definition.

The process parameters that influence the material density and defects were investigated progressively, starting from a designed workspace. The workspace was defined by the laser power (150–400 W), and laser speed (500–900 mm/s), keeping constant the layer thickness of 50 µm and hatch distance of 0.1 mm, using a 90° scanning strategy with 90° direction change between adjacent layers. Figure 3 presents the workspace definition.

The same workspace was used to manufacture specimens with a 67° scanning strategy with a 90° direction change between successive layers. Based on the results obtained during these experiments, a preliminary parameter set was defined, and further parameter fine-tuning was made to select appropriate layer thickness and hatch distance values.

### 2.2. Density and Porosity Measurement

All specimens were mechanically removed from the building plate, and the support material was removed by grinding and polishing. The bulk density of the specimens was measured by the Archimedes method, according to ISO 3369 standard [37], using an analytical balance Ohaus Pioneer PX224 (accuracy of 0.0001 g) with a density determination kit for non-floating solid specimens. The auxiliary fluid used for the measurements was 99.3% purity ethanol (SC Chimopar Trading SRL, Bucharest, Romania) with temperature-known density variations. The density kit ensures the automatic calculation of the specimen’s density considering the temperature variation of the auxiliary liquid (variation with 0.1 °C, between temperatures of 10 °C and 30.9 °C), specimen’s mass weighted in air and in liquid, and air buoyancy. Therefore, the balance software uses Equation (3) for the density calculation:

ρ = [A/(A − B)] × (ρ_0_ – ρ_L_) + ρ_L_ [g/cm^3^] (3)

where: ρ is the specimen’s density, A is the mass of the specimen weighted in air, B is the mass of the specimen weighted in the auxiliary liquid; ρ_0_ is the density of the auxiliary liquid and ρ_L_ is the air density.

All specimens were cleaned and degreased with ethanol before each density measurement. According to the standard [37], for specimens greater than 5 g, the repeatability interval is 0.025 g/cm^3^, the reproducibility interval is 0.03 g/cm^3^, and the results should round up to 0.01 g/cm^3^. In this study, the average bulk density was calculated based on three measurements made on each specimen. Each bulk density value was reported using four decimals, and if there were differences higher than 0.0025 g/cm^3^ between values, two more measurements were made. The densification degree of additive manufactured materials is usually reported by the relative density, and therefore, a reference value for a fully dense material is required.

In a very recent paper, Tarasov et al. [38] have shown that so far, no unified and accurate method has been proposed for calculating the density of heat-resistant nickel alloys. This paper reviews some of the available approaches to assess the density of alloys and proposes a new formula to calculate the density of Ni-based alloys with higher accuracy based on the chemical composition. Although their formula has been validated for several Ni-base alloys, all these alloys have a high content of gamma prime forming elements (Al and Ti) and do not contain iron, which is not the case of the investigated IN 625 alloy.

For this study, an analytical method was preferred to calculate the theoretical density of IN 625 based on the chemical composition of the powder batch presented in Table 1 and the densities of the alloying elements.

The calculations led to a value of 8.49 g/cm^3^ for the theoretical density of the investigated alloy. The theoretical density was used as a reference to calculate the relative density as the degree of densification of the manufactured material.

The material’s density is correlated with the material’s porosity, thereby a porosity analysis was performed.

**Figure 4:**
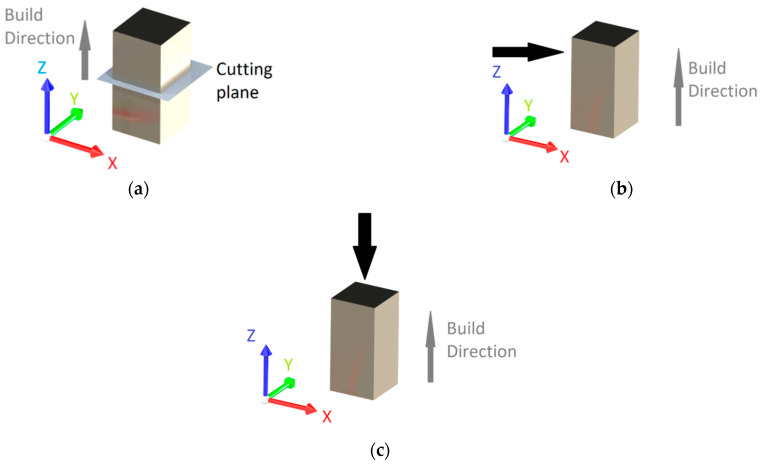
Workspace definition indication of surface for porosity measurements (**a**), roughness measurements (**b**), and top surface morphology investigation (**c**).

Porosity analysis was assessed using light optical microscopy images made at lower magnification (100×) using a Zeiss Axio Vert.A1 MAT microscope. The images were taken on metallographically prepared, unetched specimens. A binarization technique was achieved by adjusting the brightness and contrast of the optical microscope images to highlight the pores. The technique consisted of converting the images in a 16-bit grayscale format followed by conversion into black and white threshold images. At this point, white represents homogenous material, while black represents the pores. For porosity quantification, Scandium software was used. The porosity was measured in the specimen’s cross-section for the entire specimen batch manufactured with the scanning strategy of 67°, as schematically shown in Figure 4a). Measurements were performed for each specimen on 10 micro-areas of 3.5 mm^2^ each.

### 2.3. Roughness Measurement

The as-built specimens’ Ra roughness was measured using a MarSurf PS 10 mobile roughness measuring instrument with an evaluation length of 12.5 mm according to the ISO 4288 [40] standard. The measurements were performed on specimens’ sides (ZY plane), as shown in Figure 4b. The top surface morphology investigation was performed by scanning electron microscopy using the SEM FEI Inspect F50, on the specimen’s face marked with a black arrow in Figure 4c.

## 3. Results

### 3.1. Process Parameters Initial Optimization

**Figure 5:**
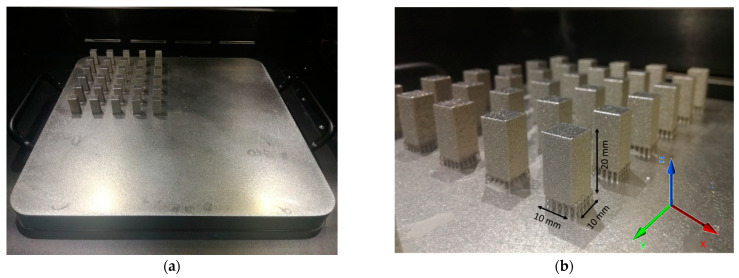
First batch of PBF-LB manufactured IN 625 specimens: (**a**) specimens on the building plate; (**b**) orientation and dimensions of the specimens.

The initial optimization of process parameters was made considering variable laser powers and laser speeds. Prismatic specimens were manufactured according to a workspace defined by laser power and laser speed, while keeping constant the layer thickness, and hatch distance, and using two scanning strategies. Figure 5 presents the first batch of PBF-LB manufactured specimens, using the 90° scanning strategy.

**Figure 6:**
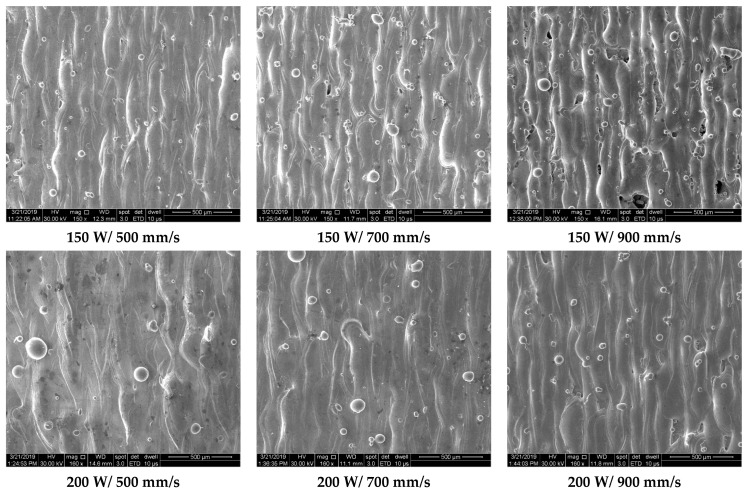

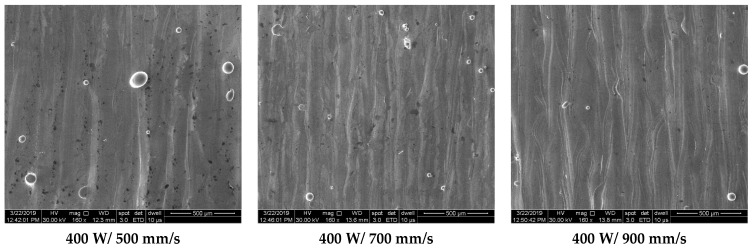
Influence of laser power and laser speed on balling effect (90° scanning strategy).

Figure 6 presents SEM images with the top surface of built specimens, using a 90° scanning strategy. The specimens’ top surface morphology showed the influence of laser power and laser speed on the balling effect. The effect is pronounced at low laser powers and laser speeds, and it decreases as the power increases.

The morphological investigation has also revealed that the lowest power levels (150 W and 200 W) and increasing the laser speed led to LOF defect formation. In the case of the 900 mm/s laser speed, at lower laser powers, LOF defects were observed, the effect being more pronounced for the lowest laser power. By studying the LOF defects according to the VED values, the SEM analysis highlighted that the best results were obtained within the VED range of 65–90 J/mm^3^.

**Figure 7:**
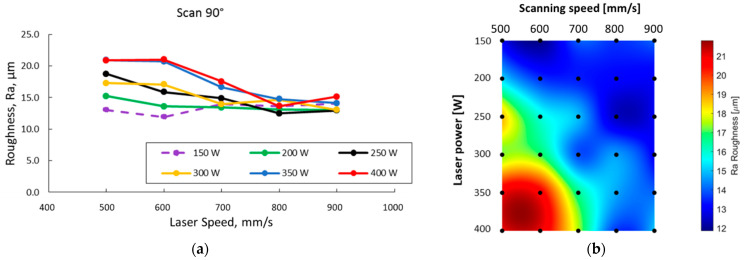

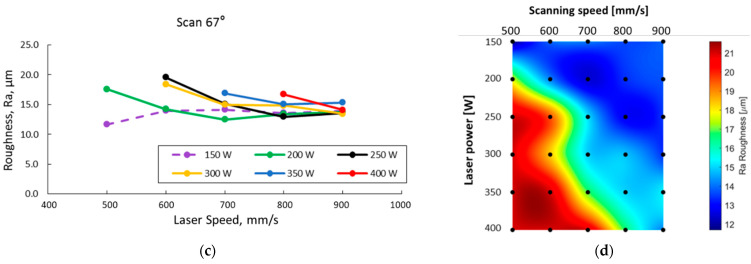
Specimen sides’ Ra roughness as a function of laser speed for 90° scanning strategy (**a**) and 67° scanning strategy (**c**), and the corresponding laser power–scanning speed roughness maps for 90° scanning strategy (**b**) and 67° scanning strategy (**d**).

The Ra roughness determined on the ZY surface was within 12–21 μm. The surface roughness measurements on specimen sides have highlighted that the roughness increases as the laser power increases and the laser speed decreases. The scanning strategy also influences the surface roughness. Figure 7 presents the surface roughness variation with laser speed at different laser powers for the two scanning strategies. Moreover, to highlight the evolution of roughness over the workspace, roughness maps were realized for both scanning strategies used.

The specimens manufactured with a 67° scanning strategy had a measurable Ra within the range of 12–20 μm, but it was noticed that high energy, high laser power, and low laser speeds generate particle agglomerations of different sizes on the specimens’ side surfaces thereby the surface roughness is out of the measuring range. An excessive increase in VED at lower laser speeds causes poor surface roughness.

**Figure 8:**
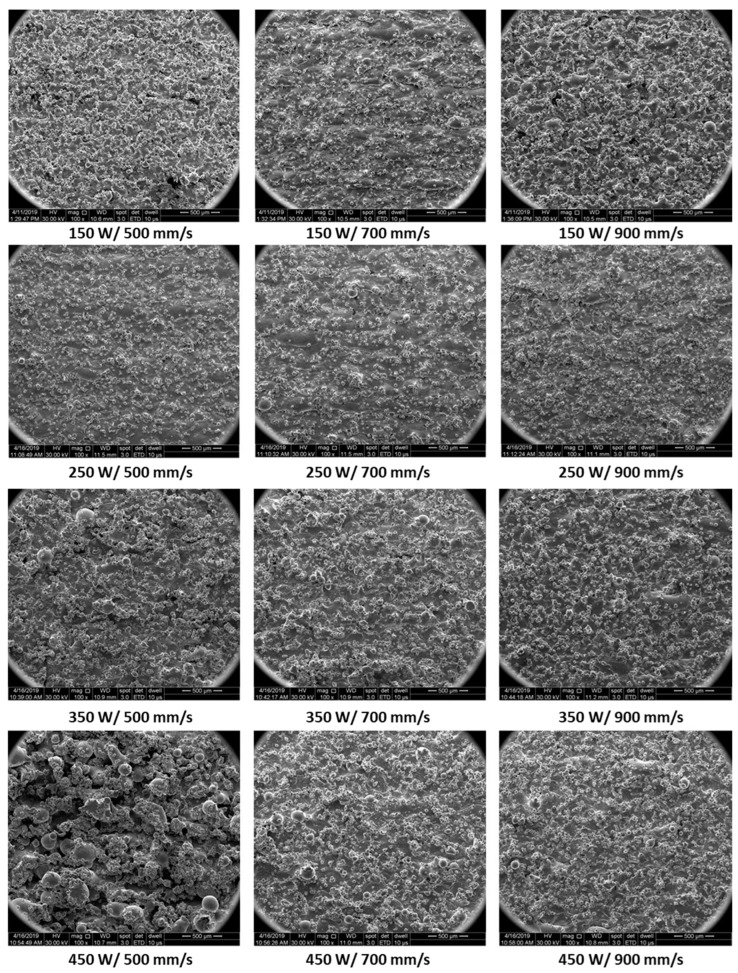
Surface morphology of specimens’ sides for low, medium, and high laser power and 500 mm/s, 700 mm/s, and 900 mm/s laser speeds (67° scanning strategy).

Such particles may occur either by liquid metal sputtering at high power and low speed or by sticking metal powder particles from the powder bed by the liquid pool, or both. The SEM images presented in Figure 8 compare the side surface morphology for low, medium, and higher laser powers at different laser speeds, respectively. SEM images were taken on the same specimens’ sides for roughness measurements.

**Figure 9:**
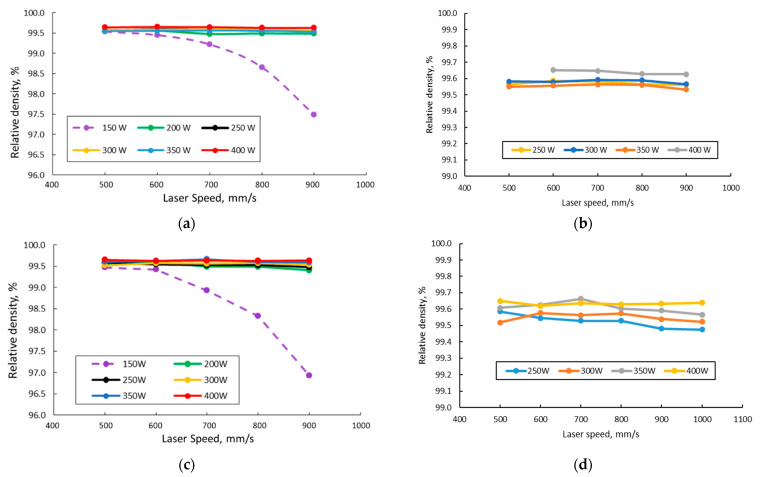
Relative density as a function of laser speed for different laser powers for 90° scanning strategy (**a**,**b**), and 67° scanning strategy (**c**,**d**).

The specimens’ density measurements showed that in both cases (90° and 67°scanning strategy), the density generated at lower laser powers (using 150 W and to a good extent 200 W) sharply drops as the laser speed increases. Images from Figure 9a,c present the relative densities of all specimens as a function of laser speed for different laser power levels, while the images from Figure 9b,d present the relative densities for the specimens manufactured with laser powers over 200 W.

As a general observation, for laser powers above 200 W within the experimental range, the IN 625 relative density increases as the laser power increases and tends to decrease with an increase in laser speed. Experimental results showed that for the specific alloy and process parameters used, laser power and laser speed play a determining role in producing a relative density higher than 99.5% of the theoretical density.

**Figure 10:**
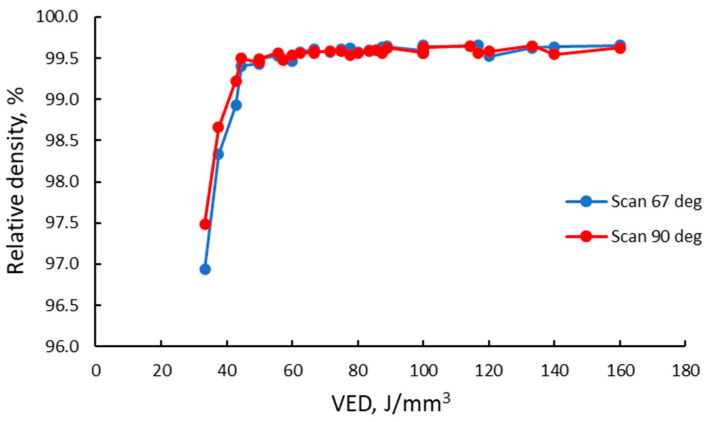
Relative density vs. VED for 90° and 67° scanning strategy.

The analysis of the relative density of the as-built alloy as a function of VED (Figure 10) gave evidence that within the laser power–laser speed experimental workspace, the relative density increases rapidly from the lowest VED value until a value of 45 J/mm^3^. Furthermore, it increases steadily at a lower rate. It can be observed that the VED values between 60–100 J/mm^3^ generate consistent relative densities of approximately 99.60% for the 90° scanning strategy and 99.58% for the 67° scanning strategy. For higher VED values, the results are more spread.

This behaviour may be caused by high energy (high laser power and low laser speed) generated material inhomogeneities mainly by gas entrapment and “keyhole” type porosity formation. At high energies, material inhomogeneities can form due to the changes in the melt pool’s dimensions, flow, and stability. When high laser power and low laser speed are used, the melt pool shape is changed from semi-circular to a longer “comet-like” form [41], and it becomes irregular or discontinuous and unstable [42], resulting in defects like porosities, spattering, and balling. These defects can be explained by the Kelvin–Helmholtz hydrodynamic instability or the Plateau Raleigh capillary instability [1].

The careful observation of the manufacturing process highlighted several aspects that affect the production of a high-quality alloy. First, at high laser powers (350 W and 400 W), a large amount of smoke is generated at almost all laser speeds. The smoke production is more pronounced at lower laser speeds. Secondly, at lower laser speeds (500 mm/s and 600 mm/s) and high laser powers, the liquid pools are pulverized, and spatters are projected randomly on the top surface of the built solid parts and on the powder bed.

**Figure 11:**
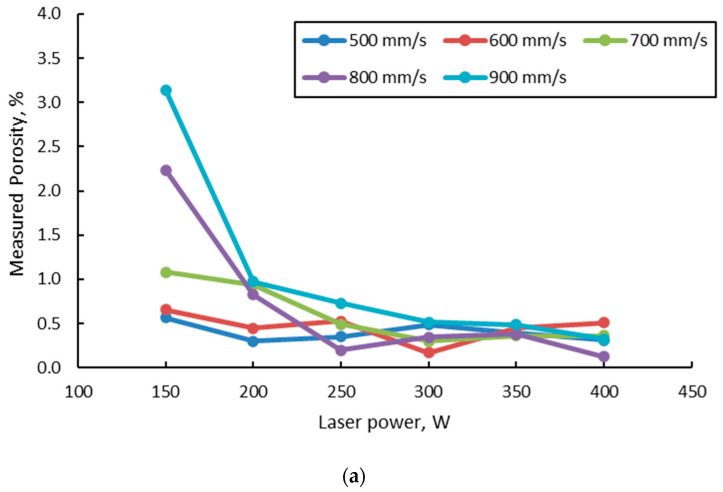

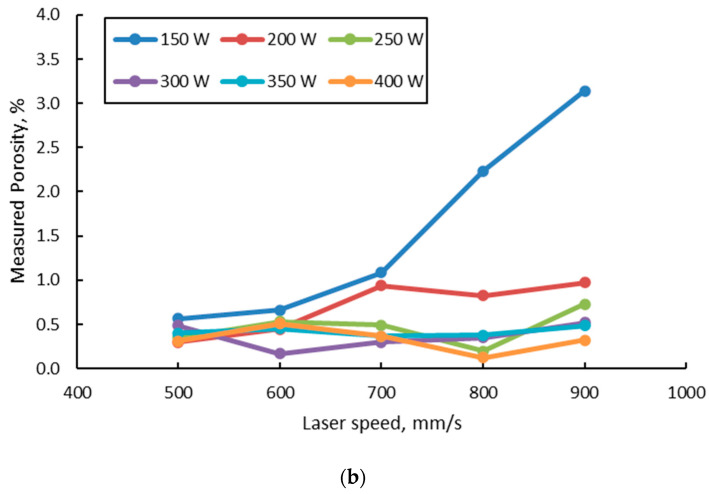
Measured porosity vs. laser power at different laser speeds (**a**) and vs. laser speed for different laser power (**b**).

For a more detailed analysis, porosity measurements were done on the entire batch of specimens manufactured with a 67° scanning strategy. Figure 11 shows the measured porosity as a function of laser power and laser speed.

**Figure 12:**
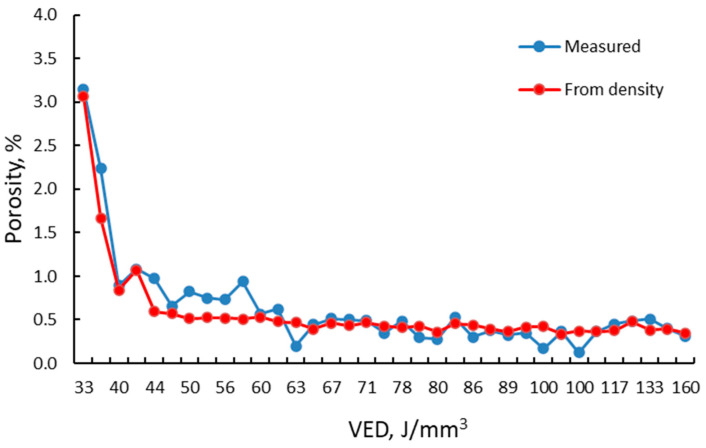
Measured and calculated porosity vs. VED.

The results from Figure 11 show that the laser power strongly influences reducing the porosity level as the power increases. At low laser power, the increase in laser speed leads to a sharp increase in porosity, mainly due to the LOF. The increase in laser speeds for the other laser power levels still generated an increase in measured porosity, but to a lesser extent. This can be explained by the mechanism of pore formation. For all low laser powers and laser speeds, porosity is generally generated by incorporating gas bubbles (keyhole porosities) as the AM process implies the use of an inert atmosphere (argon), which is insoluble in the liquid alloy, while at high speeds, the porosities are generated because of lack of fusion, as the melt pool is characterized by an insufficient penetration depth. A comparison of the porosity level in the samples microscopically measured and calculated from density results was made as a function of VED (Figure 12).

From Figure 12, it can be noticed that between the metallographically measured porosity and that based on the relative density calculated from densities measured using Archimedes’ method, there are significant differences. No direct correlation can be done between the porosity levels measured/calculated using the two methods.

The metallographic method provides information on the density of specimens in the cutting plane and selected micro-areas. The result may differ for other measurements a few hundred microns below or above this plane. Furthermore, small scratches and indentations of the abrasive used for specimen preparation can be quantified as porosity.

**Figure 13:**
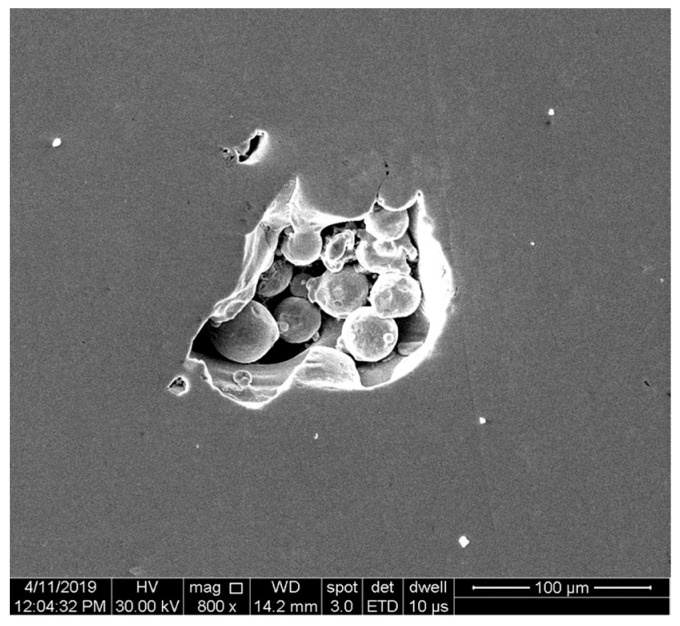
SEM image of entrapped un-melted powder in a specimen manufactured with 150 W laser power and 900 mm/s laser speed.

The porosity calculation based on the density measurement by Archimedes’ method also considers the possible entrapped un-melted metal powder in the pores resulting from the LOF. Figure 13 presents the SEM image of a pore filled with un-melted powder on a sample manufactured with 150 W and 900 mm/s laser speed. Meanwhile, the porosity calculated using the Archimedes method might be overestimated due to the simplistic theoretical density calculation. Simple density measurement will not give any information on the morphology of the porosity mode of formation (keyhole porosities, LOF, etc.). However, Archimedes’ density can provide a rapid assessment of a specimen concerning the porosity level. However, different porosity measurement methods must be conducted properly, and different methods must be used for proper equivalence [43].

**Figure 14:**
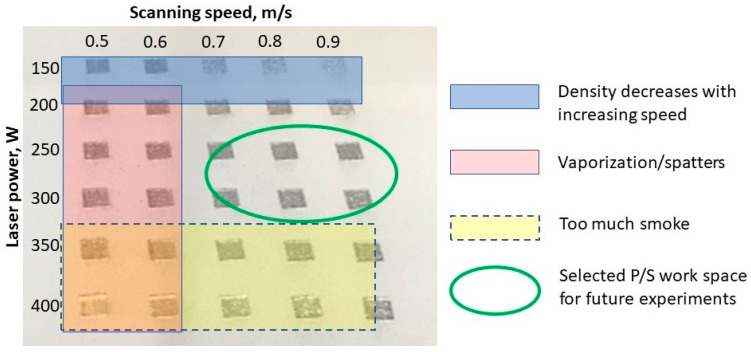
Laser power/laser speed workspace optimization.

To manufacture as densely as possible high-quality IN 625, free of contamination, the laser power/laser speed workspace was optimized considering the above density measurement results and the need to eliminate or to reduce as much as possible process sources of contamination. As is schematically presented in Figure 14, this workspace must fulfil several conditions simultaneously:

- Avoid or reduce as much as possible the smoke production and spatters.
- Avoid or reduce the alloy vaporization.
- Avoidance of those parameter sets that generate a sharp decrease in density and lack of fusion.

Based on the previous density measurements and the process constraints regarding cross-contamination of parts and/or the powder bed, the appropriate workspace for IN 625 was considered for 250 W/300 W laser power and laser speeds greater than 700 mm/s.

### 3.2. Process Parameters Fine-Tuning

#### 3.2.1. Layer Thickness

For the assessment of layer thickness influence on material density, the following process parameters were selected: laser power 250 and 300 W, laser speeds 700 mm/s, 800 mm/s, and 900 mm/s, scanning strategy 90° and 67°, layer thickness 30 µm, 50 µm, and 70 µm, hatch distance constant 0.1 mm.

**Figure 15:**
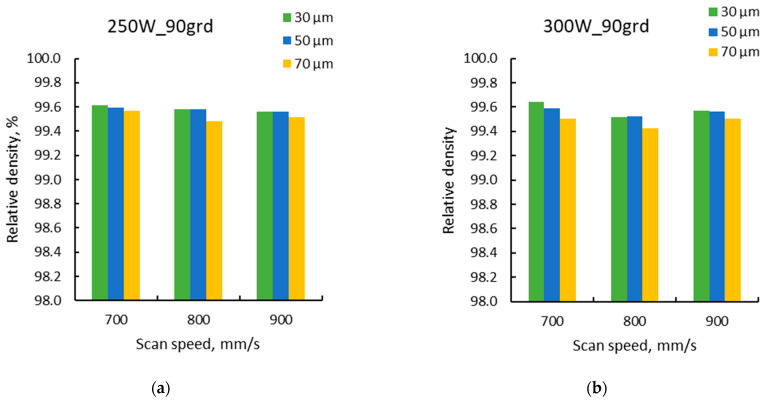

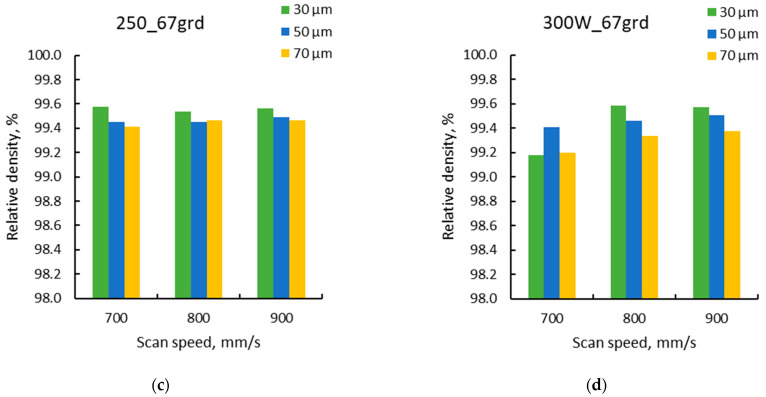
Relative density vs. laser speed for three levels of layer thickness (30 µm, 50 µm, and 70µm) and laser power powers of 250 W and 300 W for the 90° scanning strategy (**a**,**b**) and 67° scanning strategy (**c**,**d**).

The results were analysed along with those previously obtained for a layer thickness of 50 µm for both scanning strategies. The relative densities obtained as a function of laser speed for the two levels of laser power and the three-layer thicknesses (30 µm, 50 µm, and 70 µm) are presented in Figure 15 for both scanning strategies.

During the manufacturing process, higher contamination of the specimen manufactured with 300 W laser power, 700 mm/s laser speed, and 67° scanning strategy was encountered, so the density measurements were doubtful. For this reason, to have an appropriate image of the influence of layer thickness, this specimen was excluded from the analysis. All other results clearly indicate that the density decreases with increasing the layer thickness and is more accentuated at the largest layer thickness for both scanning strategy and individual laser power.

The results show that most of the results recorded for the 90° scanning strategy are grouped over a relative density of 99.5% for VED values higher than 60 J/mm^3^. For the 67° scanning strategy, for VED values between 60–100 J/mm^3^, the relative densities are more spread below and over the dotted line. This observation is consistent with the previous findings within the laser power–laser speed workspace analysis.

#### 3.2.2. Hatch Distance

For assessing the hatch distance influence on material relative density, the following process parameters were selected: laser power 250 and 300 W, laser speeds 700 mm/s, 800 mm/s, and 900 mm/s, scanning strategy 90° and 67°, layer thickness constant 50 µm, hatch distance variable 0.09 mm, 0.10 mm, 0.11 mm, and 0.12 mm.

Hatch distance is another parameter that requires optimization since high-density material can be obtained only by limiting the porosity level. Although each new layer partly melts the previously solidified one, it will inherently remain at points where the fusion of the consecutive layers is lacking, thus generating porosity.

**Figure 16:**
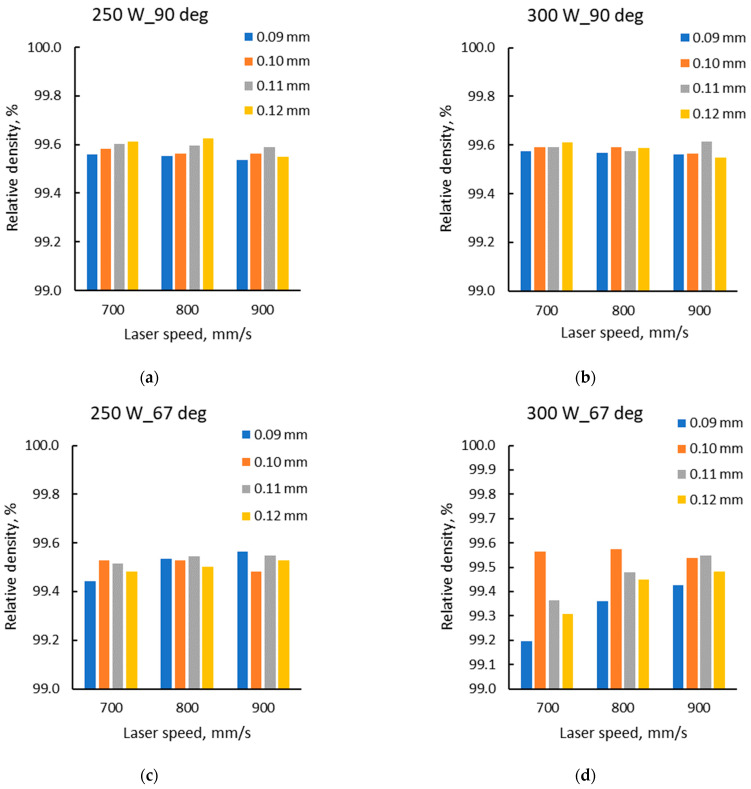
Relative density vs. laser speed for different hatch distances for as-built specimens manufactured with 90° scanning strategy (**a**,**b**), and 67° scanning strategy (**c**,**d**) respectively.

Three additional jobs were performed using hatch spacing of 0.09 mm, 0.11 mm, and 0.12 mm. The density measurements were completed with the results for the 0.1 mm hatch distance used in the first experiment. Figure 16 presents the relative density calculated for the four levels of hatch distance as a function of laser speed for the two laser powers and scanning strategies.

The general trend indicated more clearly for the specimens built with a 67° scanning strategy is that the relative density increases with the increase of hatch distance until a certain level, and then it tends to decrease with the further increase of the hatch distance.

## 4. Discussions

The PBF-LB process parameters’ influence on the densification and surface quality of IN 625 Ni-based superalloy produced using the Lasertec 30SLM machine was assessed using an iterative method, starting by defining a workspace characterized by 6 laser power values (between 150–400 W) and 5 laser scanning speeds (500–900 mm/s), by keeping constant at layer thickness of 50 µm and hatch distance of 0.1 mm, and using two scanning strategies (the 90° and 67° scanning strategy with 90° direction change between adjacent layers). Based on the first results obtained, the parameter tailoring was continued by studying the influence of layer thickness and hatch spacing.

A similar approach was used by Yang et al. [44] for the IN 718 superalloy. In their work, various process windows were studied to assess the parameters that ensure the best characteristics of IN 718. By tailoring the process parameters, they obtained a relative density above 99.9%.

To evaluate the material’s density, the Archimedes and micrographic methods were used. The most common approaches used to determine the density of additive manufactured materials are the Archimedes method, micrographic approach and X-ray scanning [45].

The study made by Spierings et al. [45] showed that the Archimedes method shows very high accuracy and repeatability of measurement, while the micrographic approach is not so accurate. Good results were also obtained in the case of X-ray scanning, including the distribution size and form of the pores. The micrographic approach is limited, as it allows the density assessment only on specific planes, while the Archimedes method allows the density assessment of the whole specimen’s volume. Moreover, Sufiiarov et al. [46] showed no correlation between the density obtained using the Archimedes method and the micrographic approach in the case of additive manufactured IN 718.

### 4.1. Specimens’ Surface Quality

The first specimen batch was manufactured using the 90° scanning strategy and a surface analysis was made, it highlighted that the combination of low laser powers and laser speeds ensured a pronounced balling effect on the top surface of the specimens. This effect decreases as the laser power increases. In the case of 150 W laser power, the best results were obtained for the lowest scanning speed, this combination ensuring a sufficient penetration depth of the laser, good wettability of the substrate and a continuous scanning track. While increasing the scanning speed, the wettability of the substrate decreases progressively, and spherical balls are formed, until they start to change into ellipsoidal formations, discontinuous tracks that led to the LOF appearance. Similar results were also observed for the 200 W laser power, but to a lower extent. These observations were supported by SEM images. Such defects were also registered by Darvish et al. [47]; they observed that low laser powers (180 W) causes insufficient overlap of the melting track while increasing the laser power causes better overlapping track coverage. Moreover, 300 W powers cause the formation of large size spatters, which was observed in this study as well. Studying the LOF defects according to VED highlighted that the lowest LOF degree was obtained within the VED range of 65–90 J/mm^3^. Amirjan et al. [48] also observed LOF defects in the cases where the process parameters did not ensure a melt pool had sufficient depth.

The specimen’s surface quality was also investigated on the side faces. The SEM images obtained in these areas showed that in the cases of low and medium laser powers, the balling effect is present, meaning small spherical balls, but to a lower extent than in the case where high laser powers are used. High energies consisting of high laser power and low laser speeds ensure the generation of the highest material inhomogeneities on the side surfaces. This is caused by changes in the melt pool’s stability, the material tends to boil and produce high sputter that solidifies at the lateral surfaces of the specimens. These defects can be explained by the Kelvin–Helmholtz hydrodynamic instability or the Plateau Raleigh capillary instability [1]. In the case of high laser powers, by increasing the scanning speed the balling is reduced, both in dimension and degree.

In the case of these prismatic specimens where the stair-stepping effect is not observed, the balling formation and LOF defects are surface defects that cause surface roughness—this defect was measured using the stylus-type method. It was observed that not only the laser power and scanning speed influence the roughness and the scanning speed. In the case of the specimens’ batch manufactured using a 90° scanning strategy, Ra values were within the range of 12–21 μm, while the 67° scanning strategy generated more agglomerated particles and the recorded Ra was between 12–20 μm. In the case of the specimens manufactured with the 67° scanning strategy, for high VED values, the surface roughness was out of the apparatus range and could not be measured.

In the SEM images, graphics, and maps, it was observed that for both scanning strategies, the high VED values (high laser powers and low scanning speeds) ensure an increased roughness. As previously explained, it is caused by the instability of the melt pool that produces a high degree of defects. Analyzing the data as a function of VED, it was observed that in the case of the batch manufactured with the 90° scanning strategy, the highest Ra values were recorded for VED values above 80 J/mm^3^, while at lower VED values, no association could be made with the Ra value recorded. For the 67° scanning strategy, the lowest Ra values were recorded for VED values under 70 J/mm^3^. Koutiri et al. [49] studied the roughness of additive manufactured IN 625 and observed that the laser power and building angle have a great influence on the material’s roughness, but they sustain that the association between the roughness and VED is unsatisfactory when considering different laser spot diameters. An increase in roughness caused by balling and defect formation was also registered by Ni et al. [50].

As was observed in the current study, the great influence of process parameters on surface roughness was also observed by Charles et al. [51] and Mumtaz și Hopkinson [28], the highest roughness being a result of different energy levels absorbed by the powder bed and different roughness values could be obtained by process parameter optimization. Similar Ra values (1–20 μm) were registered by Safdar et al. [52] due to the process parameters tailoring. In their case, the Ra values increase along with the layer thickness and beam current, and it decreases with an increase in offset focus and scan speed.

Surface roughness could be improved by applying a contour function that allows the remelting of the surface layers of the specimen; for example, Wang et al. [53] applied different contour strategies to study the influence on the Ra. They also observed the appearance of spattering for the specimen manufactured with the highest energy density (laser power 400 W and 500 mm/s scanning speed), as was registered in the current study. Another solution to improve the surface finish would be applying a laser treatment. Genna et al. [54] studied the laser finishing of EBM manufactured Ti-6Al-4V. In the as-built state, they obtained irregular surfaces with high peaks and valleys with a Ra value of 27 μm. After laser treatment, an 80% reduction in Ra was recorded. The laser treatment ensured a melting and/or vaporization of the highest peaks observed. Another solution to improve the surface roughness is to use a contour function.

### 4.2. Material Densification

The sputter formed because of the different parameter combinations affect not only the external quality of the material but also the internal quality. The LOF between layers ensures the gas entrapment and formation of porosities. It was observed that at low laser power and high scanning speed, un-melted powder particles were present. Here, the scanning speed is too high to provide sufficient time to melt the powder as the laser power is low and cannot penetrate all the powder layers. Another harmful case is when the laser power is too high, and the scanning speed is too low, the melt pool elongates and becomes unstable, the high energy of the laser melts the material and it can even boil causing evaporation of alloying elements, spattering and keyhole porosities.

Density measurements of the specimens showed that the relative density generated at lower laser powers sharply drops as the laser speed increases. For the laser powers above 200 W, within the experimental range, the alloy relative density increased as the power increased and tend to decrease with the increase in laser speed. This result is consistent with what Amirjan et al. [48] concluded in the case of SLM manufactured IN 718.

Experimental results showed that a densification degree of more than 99.5% was determined from the theoretical density in the case of IN 625. In this case, the maximum values for the as-built material were considered satisfactory. Analyzing the relative density as a function of VED showed that in the designed workspace, the relative density increases rapidly from the lowest VED value until a value of 45 J/mm^3^. Furthermore, it increases steadily at a lower rate. It can be observed that the VED values between 60–100 J/mm^3^ generate consistent relative densities of approximately 99.60% for the 90° scanning strategy and 99.58% for the 67° scanning strategy. For higher VED values, the results are more spread due to material inhomogeneities, all caused by changes in the stability of the melt pool. When high energy values are used, the melt pool size increases, while as the hatch distance increases, the width and depth of the melt pool decrease [55]. The material inhomogeneities are also influenced by the heat transfer, surface tension, and melt pool flow. In some cases, the Marangoni effect along with the recoil force cause the melt pool to become unstable and to sputter [1].

The results obtained in the case of density measurements were sustained by experimental porosity measurements.

Further, it was observed that not only the laser power, laser speed and the scanning strategy influence the material densification level, but also the layer thickness. The density decreases with the layer thickness increase and this behavior is more accentuated at the largest layer thickness for both scanning strategies. The reason for this decrease is obvious that as the layer thickness increases, in some cases, the laser power is not high enough to completely penetrate the layer, or the laser speed is too high to allow the laser to completely penetrate or melt the powder layer.

The hatch distance is another important parameter in AM processes. It should be high enough to ensure a limited overlapping of adjacent tracks, without the formation of cavities between layers or too much overlapping of the tracks that could result in other defects caused by the uneven distribution of the following powder layer or blocking of the powder coater. The results obtained for the hatch distance tailoring are somewhat spread compared to the other parameters. More pronounced spreading occurred in case of the 67° scanning strategy and at a higher laser power (300 W). The general trend of the samples built with a 67° scanning strategy is that the relative density increases with the increase of hatch distance until a certain level, and then it tends to decrease with the further increase of the hatch distance. In the case of the 90° scanning strategy, the results show in most cases that a higher hatch distance ensures a better densification for the laser speeds of 700 mm/s and 800 mm/s. It was observed that low hatch distance mainly shows a lower densification (regardless of the scanning speed), which is believed to be caused by gas entrapment between tracks and porosity formation.

With a general conclusion regarding the influence of scanning strategy, it can be said that the 90° scanning strategy produces a lower defect percentage that is translated in a higher densification, than the 67° scanning strategy. The 67° scanning strategy is used by the researchers usually to ensure a fine microstructure compared with the microstructure formed in case of a 90° scanning strategy. All these observations are supported by the fact that in the case of the 67° scanning strategy, a higher cooling rate is registered and a higher thermal gradient, this being presented in another study by the authors [56].

A specific set of process parameters was defined at the end of this study to ensure a high-density material with acceptable surface quality. This combination of parameters was established, not only based on the experimental results obtained, but also by observing other aspects of the process that affects the production of a high-quality material. For example, the production of smoke at high laser powers was more pronounced at low laser speeds, as well as the pulverization of liquid alloy that projects spatter on the powder bed and on specimens.

This study advances the knowledge of additive manufacturing of high density IN 625 alloy, manufactured by SLM with Lasertec 30 SLM equipment. The study also provides useful information for selecting the appropriate workspace of process parameters to obtain high densification or low surface roughness or a suitable combination of the two. This basic set of process parameters was used in other studies made by the authors to assess the edge and corner effects during selective laser melting [57], the tensile strength anisotropy of the additive manufactured IN 625 using a Lasertec 30 SLM machine [56], and regarding complex-shaped parts manufacturing [58,59]. Future research will be conducted regarding the appropriate process parameter definition for Lasertec 30 SLM manufactured Ti-based alloys.

## 5. Conclusions

The goal of this study was to optimize the PBF-LB workspace defined as variables by laser power, laser speed, layer thickness, and hatch distance using two different scanning strategies to improve the densification and surface quality of additive manufactured IN 625. For this research, a workspace was designed and progressively improved until an appropriate set of process parameters was selected for producing a high density IN 625 Ni-based superalloy manufactured using the Lasertec 30 SLM machine. The results obtained revealed that the densification and surface quality of the material are influenced by defects such as LOF, porosity and roughness to different degrees as a function of process parameters combinations. The combination of low laser powers and laser speeds ensure a pronounced balling effect on the top surface of the specimens, which is reduced as the laser power increases. It was found that the scanning strategy influences the material’s roughness—values within the range of 12–21 μm were determined for the specimens manufactured with a 90° scanning strategy, while the 67° scanning strategy ensures surfaces characterized by higher roughness (out of the measuring range) in the cases where high laser power and low scan speeds were used.

From all laser power values used, the laser power of 250 W generates more consistent relative densities, regardless of the scanning pattern, layer thickness, or hatch distance values applied. The best relative densities recorded were approximately 99.60% for the 90° scanning strategy and 99.58% for the 67° scanning strategy.

Based on the experimental results, a basic set of parameters that ensure a higher relative density than 99.5% from the theoretical density was defined. The appropriate parameters consisted of a laser power of 250 W, laser speed of 750 mm/s, a layer thickness of 40 µm, and a hatch distance of 0.11 mm.

Based on the analyses performed and results obtained, it was concluded that the process parameters have a significant influence on the stability of the melt pool that causes the internal and external defects.

## Figures and Tables

**Table 1:** Actual chemical composition of the investigated alloy.

Alloying Element	Specification, wt.% [39]	Metal Powder Chemical Composition, wt.% *
Al	0.40 max	0.06
C	0.10 max	0.02
Co	1.0 max	0.1
Cr	20.0–23.0	20.7
Fe	5.0 max	4.1
Mn	0.50 max	0.01
Mo	8.0–10.0	8.9
Nb	3.15–4.15	3.77
Si	0.50 max	0.01
Ti	0.40 max	0.07
Ni	rem	rem

* Provided by the manufacturer in the powder batch test certificate.
